# Identification of Novel Immune Ferropotosis-Related Genes Associated With Clinical and Prognostic Features in Gastric Cancer

**DOI:** 10.3389/fonc.2022.904304

**Published:** 2022-05-18

**Authors:** Chen Xiao, Tao Dong, Linhui Yang, Liangzi Jin, Weiguo Lin, Faqin Zhang, Yuanyuan Han, Zhijian Huang

**Affiliations:** ^1^Department of Gastroenterology, Fuzhou Second Hospital Affiliated to Xiamen University, Fuzhou, China; ^2^Department of Digestion, Yidu Central Hospital of Weifang, Weifang, China; ^3^Graduate School of Fujian Medical University, Fuzhou, China; ^4^Institute of Medical Biology, Chinese Academy of Medical Sciences and Peking Union Medical College, Kunming, China; ^5^Department of Breast Surgical Oncology, Fujian Medical University Cancer Hospital, Fujian Cancer Hospital, Fuzhou, China

**Keywords:** immune, ferropotosis, gastric cancer, prognosis, biomarkers

## Abstract

**Background:**

Gastric cancer (GC) is the fifth commonest cancer and the third commonest reason of death causing by cancer worldwide. Currently, tumor immunology and ferropotosis develop rapidly that has made gastric cancer be treated in new directions. So, finding the potential targets and prognostic biomarkers for immunotherapy combined with ferropotosis is urgent.

**Methods:**

By mining TCGA, immune-related genes, ferropotosis-related genes and immune-ferropotosis-related differentially expressed genes (IFR-DEGs) were identified. The independent prognostic value of IFR-DEGs was determined by differential expression analysis, prognostic analysis, and univariate and lasso regression analysis. Then, based on the prognostic risk model, the correlation between IFR-DEGs and immune scores, immune checkpoints were evaluated. Besides, we predicted the response of high and low risk groups to drugs.

**Results:**

A 15-gene prognostic feature was constructed. The high-risk group had a poorer prognosis than the low-risk group. High-risk group had higher level of Treg immune cell infiltration compared with that in the low-risk group, and the tumor purity, immune checkpoint PD-1 and CTLA4, and immunity in the high-risk group were higher than those in the low-risk group. These results indicate that immune ferropotosis-related genes migh be potential predictors of STAD’s response to ICI immunotherapy biomarkers. In addition, the response of small molecule drugs such as Nilotini, Sunitinib, Imatinib, etc. for high and low risk groups was predicted.

**Conclusion:**

IFRSig can be regarded as an independent prognostic feature and may estimate OS and clinical treatment response in patients with STAD. IFRSig also has important correlation with immune microenvironment. A new understanding of the immune-ferropotosis-related genes during the occurrence and development of STAD is provided in this study.

## Introduction

Gastric cancer (GC) is the fifth commonest cancer and the third commonest reason of death causing by cancer worldwide (1), and is one of the commonest digestive system cancers (2). Frequent recurrence and metastasis contribute to the diagnosis, treatment and high death rate in GC (3, 4). However, the discomfort symptoms in the early stage of GC are less obvious or not. In most cases, GC has developed that cannot be treated surgically and radically by the time of diagnosis. Tumor pathology (T), lymph node biopsy (N) and distant organ metastasis (M) are currently the main criteria for judging patients’ prognosis, but there are also great differences in the prognosis of patients under the same TNM stage (5).

Ferroptosis, as a newly regulated cell death mode, differs from the molecular characteristics of other forms of regulatory cell death. Its genetic, biochemical and morphological characteristics are classified as iron-dependent cell death and superoxide lipids Accumulation (6). Ferropotosis is closely associated to tumor occurrence and development. Some studies have shown that ferropotosis-related factors or pathways can regulate the sensitivity of tumor cells to ferropotosis by affecting related mechanisms such as iron metabolism, ROS synthesis, and antioxidant system. It is reported that ferritin phagocytosis is mediated by interacting with surface arginine residues in ferritin heavy chain 1 (FTH1) (7, 8). NCOA4 overexpression induces ferropotosis by increasing intracellular free iron content, glutathione production, and reactive oxygen species (ROS) levels (9). Ferropotosis plays a vital role in varied diseases, which include GC (10–12). Targeted ferropotosis may be a potential therapeutic strategy for GC patients. Over the years, according to the observation, the prognosis of GC is related to pathological staging, moreover, tumor immune status may impact the prognosis of patients highly (13). Closely related to the development of tumor immunosuppressive microenvironment, A large number of studies on the relationship (14, 15). The pathogenesis of GC has been newly recognized through a large number of studies on the relationship between iron death and immunity, including the intervention of ferropotosis can effectively improve immune suppression (16, 17).

As lots of ferropotosis-related genes regulating the relation between ferropotosis and tumor, the prognosis of patients can be evaluated by the expression of ferropotosis-related genes in tumor tissues. In this study, the STAD gene expression information was obtained by the analysis of Cancer Genome Atlas (TCGA) database, and then the differential expression of immune ferropotosis-related genes in the sample was analyzed, thus, the survival of STAD patients was predicted effectively by constructing a model containing multiple genes. The correlation between the risk scoring model and immune status was analyzed, the potential mechanisms were explored, diagnosis and treatment basis for clinical treatment were provided, and new therapeutic targets were found.

## Materials and Methods

### Microarray Data Analytics and Screening of Differentially Expressed Genes Related to Immune Ferropotosis

To compare differentially expressed genes (IFR-DEG) associated with immune ferropotosis in STAD, TCGA database was used for subsequent analysis. The downloading of human immune-related genes (IRGs) from the ImmPort database (https://www.immport.org./home) and the GeneCard database (https://www.genecards.org/) reached 17,500 in total, and the downloading of ferropotosis-related genes (FRGs) from the FerrDb database (http://www.datjar.com:40013/bt2104/) and previous literature (18) reached 398 in total. The cutoff condition settings are log2 fold change (logFC) < -2, p -value < 0.05, which is statistically evident.

### Construction and Verification of a Prognostic Model of Differentially Expressed Genes Related to Immune Ferropotosis

Based on a preliminary screening of IFR-DEGs with differentially expressed, the survival-related IFR-DEGs with significant prognostic value (p < 0.05) was determined by performing a single factor Cox analysis of overall survival (OS). The predictive models of candidate immunity and ferropotosis-related IFR-DEGs were determined by performing minimum absolute contraction and selection operator LASSO proportional regression.

A total of 352 STAD patients were randomly assigned to either the training cohort or the test cohort in a 1:1 ratio to construct and validate risk scores (176 in the training cohort, 176 in the test cohort). For the training cohort, a linear combination of expression values for each prognostic gene was applied to construct the prognostic risk profile of our immune and ferropotosis-related genes (IFRSig). The establishment of profile was based on corresponding coefficients. Patients from the TCGA-STAD dataset were divided into low-risk group and high-risk group according to the median value of the risk score. Principal component analysis (PCA) was used to explore the distribution characteristics of different groups through the R package. Finally, the validity of prognostic indicators was evaluated by using the area under the curve (AUC) of the “time receiver operating characteristic curve (ROC) “.

Functional enrichment analysis of differentially expressed genes related to immune Ferropotosis in gastric cancer Gene Ontology (GO) and Kyoto Encyclopedia of Genes and Genomes (KEGG) pathway analyses were performed through the ClusterProfiler package, so that functional annotation and enrichment pathways were explored, where *p* < 0.05 indicating statistically evident differences.

### Survival Analysis and Verification

To assess the expression and prognostic value of IFR-DEGs in STAD further, a difference analysis and a prognostic analysis were performed through the “survival” package. According to the Cox proportional risk model and the Kaplan-Meier model, the risk ratio (HR) was calculated, Statistical significance was defined as p < 0.05 or <0.01.

### Clinicopathological Correlation Analysis and Column Diagram Construction

According to the “survival” package in the R software, with combination of clinicopathological features, the correlation between IFR-DEGs and clinicopathological features was analyzed. R package “rms” was used to obtain the column line diagram and calibration curve. The risk score related to the prognostic mode, as a prognostic factor, was used to assess the 1-year, 3-year and 5-year OS.

### Relationship Between Risk Score and Immune Cell Infiltration

SsGSEA and CIBERSORT R scripts were used to quantify the relative proportion of infiltrated immune cells. Spearman rank correlation analysis was used to explore the relationship between risk score values and immune-infiltrating cells. Furthermore, TIDE algorithm was used to evaluate two different tumor immune escape mechanisms using IFR-DEG markers.

### Predicting Response to Chemotherapy

The R package of pRRophetic was applied for predicting the median maximum inhibitory concentration (IC50) of common small molecule drugs. IC50 represents the effectiveness of the substance in inhibiting specific biological or biochemical functions. Wilcoxon symbolic rank test was used for inter-group differences.

### Statistical Analysis

R software (version 4.0.2) was applied for statistical analysis. Perl programming language (version 5.30.2) was applied for data processing. Multivariate Cox regression analysis was applied to assess prognostic significance. PCA was also performed using R’s ggplot2 package. Kaplan-Meier curves and logrank tests were used to analyze survival differences between the two groups. Pearson correlation coefficient test was used for gene correlation analysis; Spearman correlation coefficient test was applied for risk score and correlation analysis of immune cells and immune genes. When *p* < 0.05, the difference was statistically evident.

## Results

### Identification of Differentially Expressed Genes Related to Immune Ferropotosis in Gastric Cancer Compared With Normal Gastric Tissue

**Figure 1:**
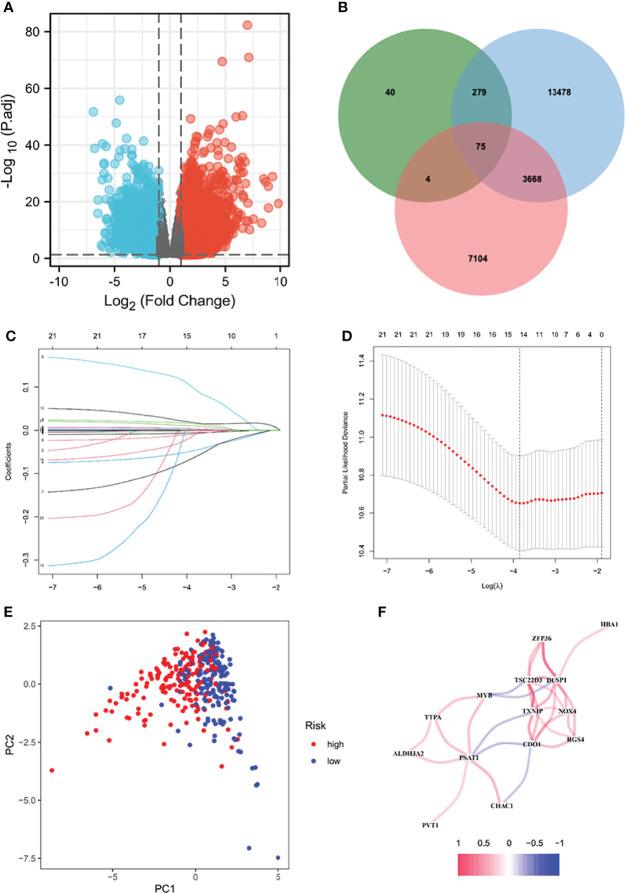
Screening for differentially expressed genes. Volcanic diagram of differentially expressed genes (DEG) between normal gastric tissue and gastric cancer **(A)**. Red represents up-regulated genes and blue represents down-regulated genes. According to three data sets **(B)** Venn diagram showing 75 immune ferropotosis genes. LASSO regression analysis of 75 DEGs. Ten-fold cross-validation was applied to calculate the best lambda, which leads to a minimum mean cross-validated error **(C)**. A total of 15 DEHGs were adopted for the LASSO model **(D)**. **(E)** PCA diagram of high and low risk groups. **(F)** Correlation network diagram of prognostic model genes. PCA, principal component analysis; IRG, Human immunity-related genes; FRG, human ferropotosis-related genes.

The volcanogram showed 7180 up-regulated DEGs and 3772 down-regulated DEGs screened in the TCGA (**Figure 1A**). Then, 17,500 human IRGs from the ImmPort database and genecard and 398 human ferropotosis-related genes from FerrDb were analyzed by Venn diagram analysis, and 75 co-expressed genes were obtained (**Figure 1B**). We used lasso regression to construct a prediction model of DEG-related risks (**Figure 1C**). The risk score formula: risk score = (Expi × βi). (Exp: model gene expression level; β: model gene coefficient) (**Figure 1D**). Coefficient risk was shown in the annex. Based on the median risk score (50%), patients had two groups: high-risk group and low-risk group. The results of PCA verify the differential expression of high and low risk groups in STAD patients (**Figure 1E**). Correlation network diagram of prognostic model genes were illustrated in **Figure 1F**.

### Construction and Verification of Prognostic Risk Model of Differentially Expressed Genes Related to Immune Ferropotosis

**Figure 2:**
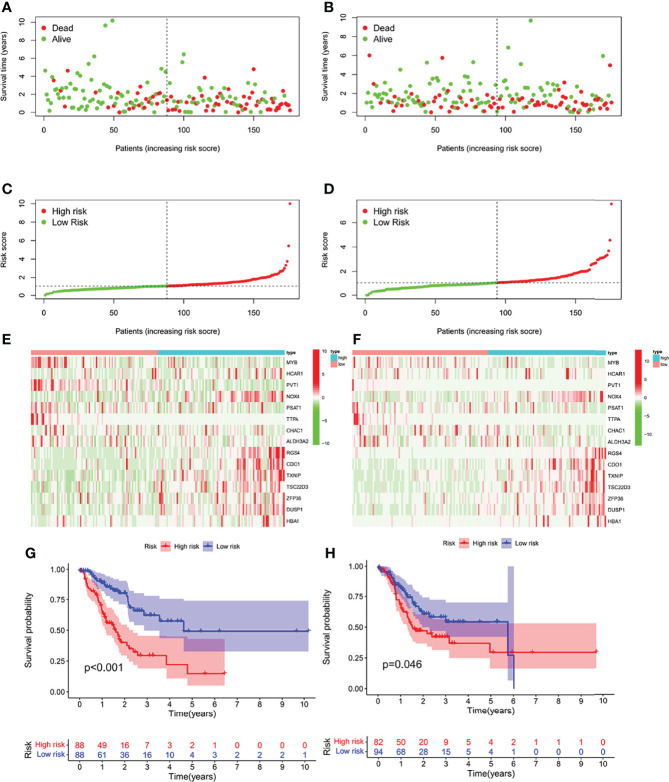
Correlation between the prognostic model and the overall survival (OS) of patients in the TCGA train Cohort **(A, C, E, G)**, the TCGA test Cohort **(B, D, F, H)**. The distribution of survival time **(A, B)**, risk score **(C, D)** and genes expression levels **(E, F)** and OS **(G, H)**. Patients were classified into low-risk and high-risk groups by using the median score as a cut-off value. The red dots and lines represent the patients in high-risk groups. The green dots and lines represent the patients in low-risk groups. STAD, gastric cancer; KM, Kaplan-Mayer; ROC, receiver operating characteristics.

According to the median risk score, high-risk group or low-risk group of patients were sorted for the training cohort and test (**Figures 2A, B**). In the training cohort, there were evident differences in OS between high-risk and low-risk groups (**Figures 2C–F**, *p* < 0.001). In the two groups of the test cohort, the same significant difference as OS was repeated (**Figures 2G, H**, *p* < 0.05). As the results showed, the clinical prognosis of patients with low-risk scores was better than that of patients with high-risk scores, which was consistent with the two groups of results in each cohort. All results showed that IFRSig might have accurate pre-measurement capabilities for OS.

### Risk Score Is an Independent Prognostic Factor

**Figure 3:**
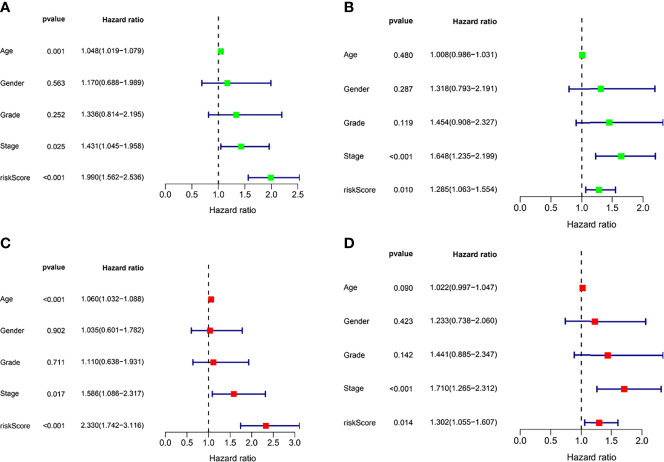
Independent prognostic factor analysis. Results of univariate Cox regression analysis to determine cancer genome mapping (TCGA) training cohort **(A)** and verify the association between overall survival and clinical characteristics in cohort **(B)**. Results of multivariate Cox regression analysis to determine TCGA training cohort **(C)** and validation cohort **(D)** correlation between overall survival and clinical characteristics in the data set.

In the TCGA cohort (training set), univariate Cox regression analysis showed age (*P* < 0.001), tumor stage (*P*= 0.025), and risk score (*P* < 0.001) had significant correlation with OS (**Figures 3A, B**), while multivariate Cox regression analysis showed that age (*P* = 8.802*e* -04), tumor stage (*P* = 0.017), and risk score (*P* < 0.001) had significant correlation with OS (**Figures 3C, D**). These results suggest that IFRGs were independent prognostic factors for STAD.

### Heat Map and GO/KEGG Pathway Enrichment Analysis

**Figure 4:**
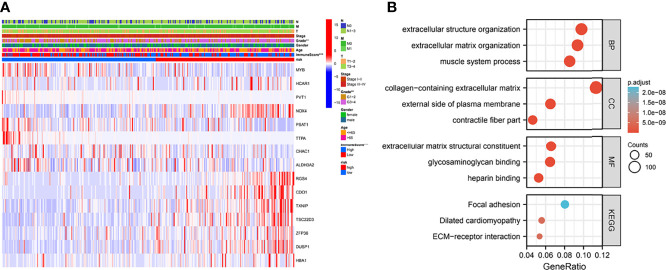
Clinically relevant heat map and GO/KEGG pathway enrichment analysis. **(A)** Based on risk characteristics associated with prognosis, a heat map using data about clinicopathological characteristics of patients was plotted. The higher the intensity of red, the higher the expression. The higher the intensity of blue, the lower the expression. **p < 0.01, ***p < 0.001. **(B)** The graph shows the GO and KEGG analysis of high and low risk differential genes.

To observe the expression of prognostic model genes in clinical features, we constructed an expression heat map based on clinical feature correlation to observe the expression relationship of prognostic model genes between high-risk and low-risk groups, as well as the patient’s age, gender metastasis, tumor stage, grade and immune score (**Figure 4A**). In addition, we found that in biological processes (BP), extracellular matrix organization, muscle system process, cellular component enriched in collagen-containing extracellular matrix, external side of plasma membrane, contractile fiber part. molecular function enriched in extracellular matrix structural constituent, glycosaminoglycan binding, heparin binding. More important, KEGG enriched in ECM-receptor interaction, Dilated cardiomyopathy, Focal adhesion (**Figure 4B**).

### The IFRSig as a Prognostic Predictor

**Figure 5:**
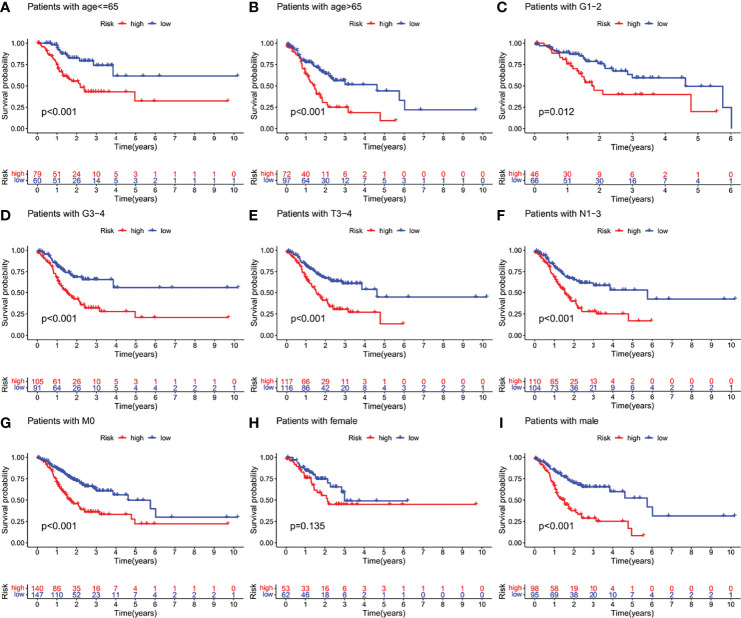
Kaplan-Meier survival curves of GC patients in different clinical subgroups. **(A–I)** OS survival curves of high and low risk GC patients in age, G1-2, G3-4, T3-4, N1-3, M0, female, male subgroups. OS, overall survival.

We examined the prognostic power of IFRSig. GC patients from TCGA cohort were reassigned age, G2-2, G3-4, T3-4, N1-3, M0, and gender as prognostic and clinicopathological factors according to different conditions. Kaplan Meier survival analysis for each subgroup and survival indicated that the operating-system GC low-risk group evidently prolonged patients regardless of age and TNM stage (P < 0.001, **Figure 5**), which suggested that IFRSig can have good predictive power in most subclinical subgroups.

### Identification and Verification of Nomograms

**Figure 6:**
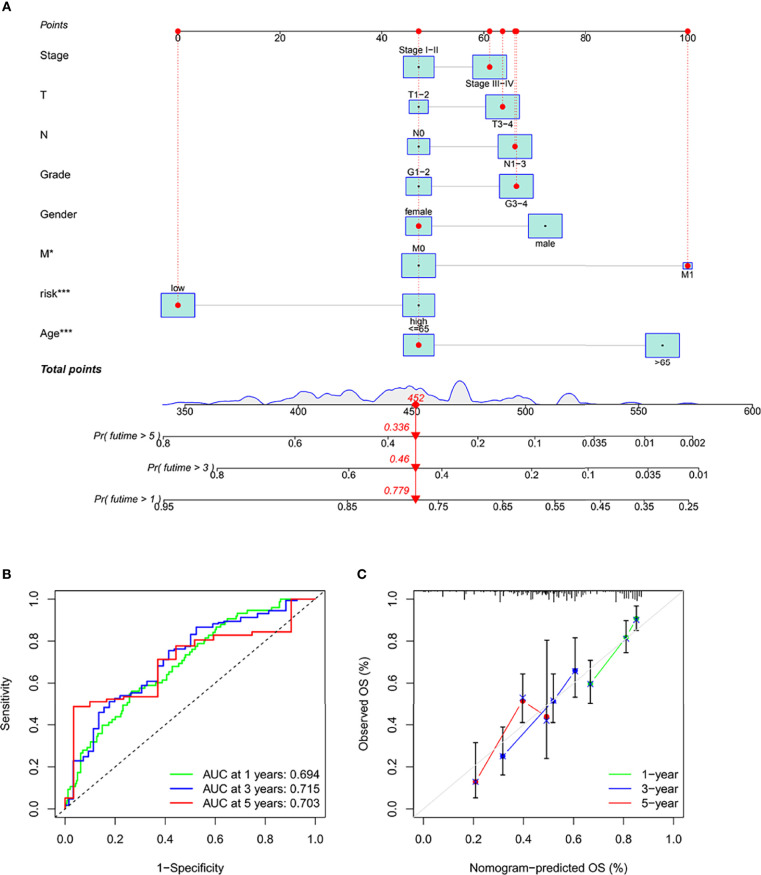
Construction and validation of a columnar chart. **(A)** Survival columnar charts based on the total TCGA cohort. **(B)** The ROC curve compares the prognostic ability of columnar charts in the TCGA cohort at 1, 3, and 5 years. **(C)** Calibration curves to predict 1, 3, and 5-year survival in STAD patients in the TCGA cohort. *p < 0.05. ***p < 0.001.

Column charts are a tool that is reliable to estimate cancer patients’ individualized risk scores. IFRSig and other clinical were used in this paper. Multivariate Cox regression analysis of pathological covariates, constructed a columnar graph based on the entire TCGA set (**Figure 6A**), in the cohort. The AUC of columnar graphs 1, 3, and 5 years were 0.694, 0.715, and 0.703, respectively, indicating that the columnar graph has good specificity and sensitivity level to OS (**Figure 6B**). The calibration chart was consistent with the diagonal, confirming the predicted value of the prognostic columnar chart for 1-year, 3-year, and 5-year OS (**Figure 6C**). All results showed that the columnar chart constructed by IFRSig had good prognostic ability for STAD patients.

### Immune Characteristics

**Figure 7:**
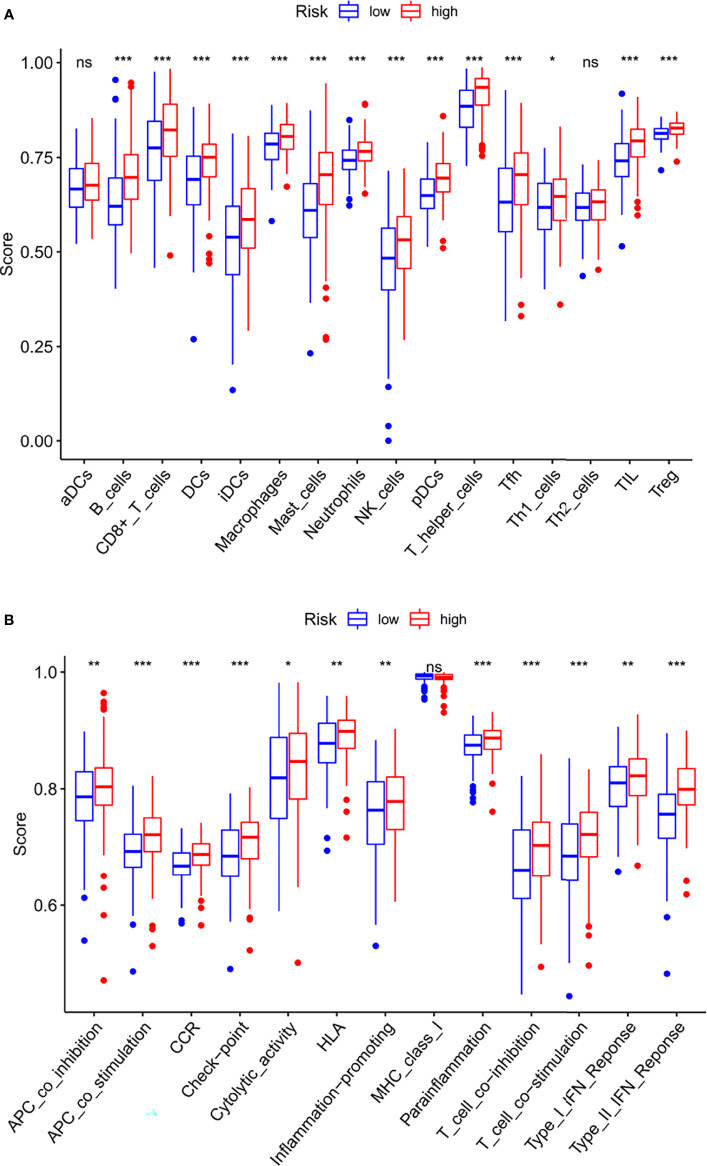
Relationship between IFRSig and immune infilration immune function. **(A)** Box plot showing association between IFRSig and Immunoinfiltrating cells. **(B)** Box plot showing association between IFRSig and immune functions. NS No Significance, *p < 0.05, **p < 0.01, ***p < 0.001.

To make the investigation of complex crosstalk between IFRSig and immune signatures better, the immunoinfiltration profile of immunoinfiltrating cells from STAD samples were assessed. We further compared the association between immunoinfiltrating cells and IFRSig, noted that regulatory T cells (Tregs, p < 0.0001) were significantly increased in STAD patients in the high-risk group (**Figure 7A**). This was in line with previous observations that link the high expression of Treg and macrophages with tumor progression and immunosuppression (19). In the correlation of immune function expression, we found that the high-risk group with functions such as check point and T_cell_co-inhibition was stronger than the lower-risk group, which indicating that the high-risk group had stronger immunosuppression (**Figure 7B**).

### The Analysis of Immune Microenvironment, Immune Escape and Immune Checkpoint

**Figure 8:**
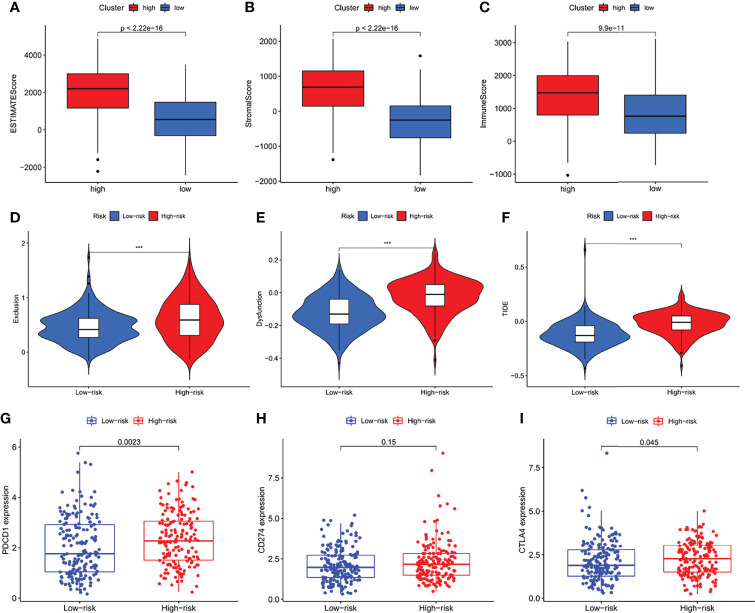
The analysis of immune microenvironment, immune escape and immune checkpoint. **(A–C)** Comparison of ESTIMATE score, Stromal score and Immune Score between high-risk and low-risk subgroups. **(D–F)** Immune Escape. **(D)** Exclusion, **(E)** Dysfunction, and **(F)** TIDE score in different risk-groups. **(G–I)** Box plot showing correlation between IFRSig and immune checkpoint (PCDC1, CD274 and CTLA-4); ***p < 0.001.

Regarding the TME score, high-risk patients had higher stromal scores, immune scores, and ESTIMATE score than low-risk patients (**Figures 8A–C**). In addition, the Tumor Immune Dysfunction and Exclusion (TIDE) algorithm was based to predict the response of risk levels to immune checkpoint inhibitors. In our results, the high-risk group had a higher Exclusion, Dysfunction and TIDE score than the low risk-group (**Figures 8D–F**). Immune checkpoints couldch regulate tumor immune infiltration, so the expression values of IFRSig were compared with 3 immune checkpoints. As shown in (**Figures 8G–I**), PD-1 (*p* = 0.0023), PD-L1 (*p = 0.15)*, CTLA-4 (*p* = 0.045) expression difference. These results suggested that the IF gene might be a potential predictive biomarker of STAD response to ICI immunotherapy.

### Potential Drugs

**Figure 9:**
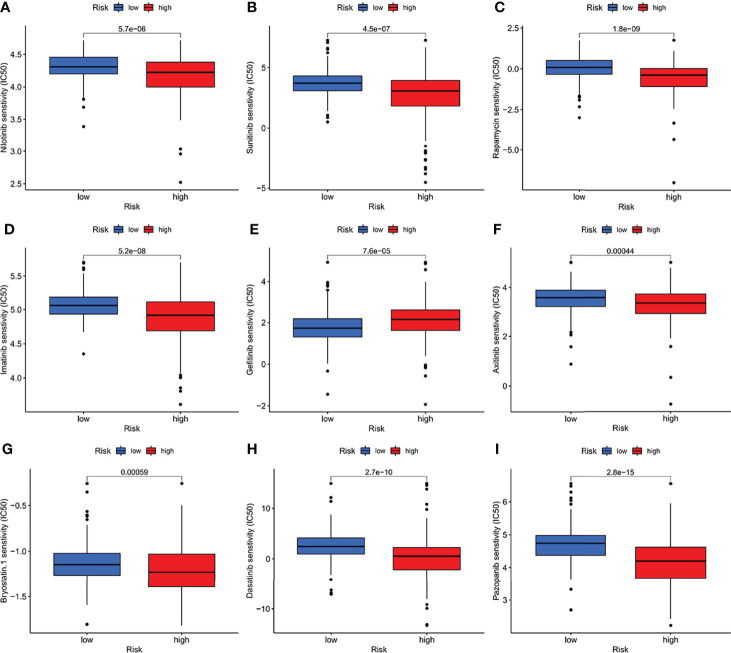
**(A–I)** The half-maximum, inhibitory concentration (IC50) of 9 common small molecule drugs (Nilotini, Sunitinib, Rapamycin, Imatinib, Gefitinib, Axitinib, Bryostatin, Dasatinib, and Pazopanib).

Many small molecule drugs were often resistant during cancer treatment, resulting in poor efficacy of drugs for gastric cancer and worse clinical results (20). To verify the use of drugs in different risk groups, the median maximum inhibitory concentration (IC50) was compared. IC50 is helpful for the quantification of the therapeutic ability of drugs to induce cancer cell apoptosis, which is inversely proportional to the sensitivity of small molecule drugs (21). Based on the pRophetic algorithm, we calculated the effects of 9 common targeted drugs for tumors (Nilotini, Sunitinib, Rapamycin, Imatinib, Gefitinib, Axitinib, Bryostatin, Dasatinib, and Pazopanib) in GC patients, and explored the relationship between risk scores and drug resistance based on IC50 (**Figures 9A–I**). Those with p > 0.05 were excluded. As **Figure 9E** showing, high-risk group has evidently higher IC50 of Gefitinib than that in the low-risk group, indicating that patients with high IFRSig may not profit by these drugs. Nilotini, Sunitinib, Rapamycin, Imatinib, Gefitinib, Axitinib, Bryostatin, Dasatinib, and Pazopanib were significantly reduced in the high-risk, group, so these small molecule drugs might be more sensitive to high-risk patients and had a greater impact. The results show that the risk prognosis model not only could divide individuals into groups with different risk, but also could assist in the selection of small molecule drugs according to the sensitivity values corresponding to clinically observed GC patients.

## Discussion

GC is a highly heterogeneous malignant tumor with an increasing incidence, poor prognosis and high mortality worldwide (22). It’s proved that commonly used clinicopathological parameters (such as TNM stage, age, gender, viral infection and serum CEA level) are not sufficient to correctly predict the prognosis of patients (23, 24). Thus, it has become an urgent clinical problem to be solved to study the mechanism of the occurrence and development of gastric cancer. We learned that ferropotosis-related genes are closely associated to cancer, and their expression levels change with cancer stages (25). Few studies have linked the prognosis and treatment of STAD with ferropotosis-related genes. Hope to illustrate this correlation with some analysis.

We performed Venn diagram analysis based on gastric cancer differential genes and immune ferropotosis related genes in TCGA. Obtaining 75 co-expressed immune ferropotosis-related DEGs, we established a prognostic risk model after performing lasso regression analysis. Then, the independent prognostic value of IFRSig was determined by differential expression analysis, prognostic analysis, and multivariate cox regression analysis. All results show that the risk model has good prognostic ability for STAD patients.

To provide new insights into the pathogenesis of STAD and an effective tool to predict STAD therapeutic effect, which may contribute to provide additional therapeutic and prognostic benefits. We assigned cancer samples to the high-risk group and the low-risk group, IFRSig, the dominant factor in the prognostic risk model and column diagram. Our results have a satisfactory correlation with clinical results, which indicates that IFRSig is a good predictor of risk factors. It is of note that this model might be further improved by more advanced machine learning algorithms as illustrated in other similar medical studies (26–28).

Immunity and ferropotosis-related genes or pathways have been shown to be involved in the proliferation, differentiation, invasion and metastasis of gastric tumors through different pathways of tumor progression and pathogenesis. At present, it has been confirmed that a variety of immune genes (29–31) have different tables in gastric cancer tissues, and participate in multiple processes of gastric cancer occurrence, including the proliferation, apoptosis, and migration of tumor cells.

In the tumor microenvironment, cancer cells and immune cells exert a large number of chemokines and cytokines to regulate the onset and progression of tumors. We studied and found that that regulatory T cells increasing is especially crucial. The strong immunosuppressive microenvironment in cancer is a key challenge for treating cancer. Tregs and tumor-associated macrophages can directly reduce T cell proliferation in the immune microenvironment. It can also affect the invasiveness of tumors by affecting lactate metabolism. Our study suggests that high-risk STAD patients may be associated with immune escape. However, the mechanism between immune ferropotosis genes and Tregs is still unclear, and it needs to research further to solve this problem. We further assessed the expression levels of these immunosuppressive checkpoint inhibitors and found that IFRSig is related to CTLA-4, PD-1, PD-L1, among which PD-1 had the greatest association. The resistance and sensitivity of small molecule drugs were analyzed to predict the potential of IFRSig to determine the therapeutic effect.

The study is not perfect enough. The predictive power of the prognostic model has not been verified in the GEO cohort and the ICGC cohort. We are also cared for whether the correlation between genes, ferropotosis and immunity can be accurately calculated by the general correlation test. In addition, the study is conducted at a bulk level, a single cell study will be more accurate in reflecting cell heterogeneity (32). This study is a retrospective analysis according to bioinformatics data and has not yet verified the prospective analysis, which needs more experimental and clinical data.

In summary, an IFRSig that is closely related to the prognosis of STAD is developed. It can better estimate OS in combination with immunological characteristics and can predict the clinical treatment response of STAD.

## Conclusion

In this study, immune ferropotosis-related genes with independent prognostic value were obtained through comprehensive bioinformatics analysis. A prognostic risk model was established. There are significant correlations between immune ferropotosis-related genes and immune scores, immune checkpoints, small molecule drugs. IFRSig can be regarded as an independent prognostic feature and may estimate OS and clinical treatment response in STAD patients. This is a new understanding of IF genes during the occurrence and development of STAD.

## Data Availability Statement

The original contributions presented in the study are included in the article/supplementary material. Further inquiries can be directed to the corresponding authors.

## Author Contributions

Author YH and ZH conceived designed the study. CX, TD, and LJ performed the experiments. WL, LY, and FZ analyzed the data. CX wrote the manuscript. All authors contributed to the article and approved the submitted version.

## Funding

This work was supported by the Natural Science Foundation of Fujian Province (No. 2020J011112) and Joint Funds for the innovation of science and Technology, Fujian province (Grant number: 2020Y9039).

## Conflict of Interest

The authors declare that the research was conducted in the absence of any commercial or financial relationships that could be construed as a potential conflict of interest.

## Publisher’s Note

All claims expressed in this article are solely those of the authors and do not necessarily represent those of their affiliated organizations, or those of the publisher, the editors and the reviewers. Any product that may be evaluated in this article, or claim that may be made by its manufacturer, is not guaranteed or endorsed by the publisher.
